# Interactive Learning: Online Audience Response System and Multiple Choice Questions Improve Student Participation in Lectures

**DOI:** 10.7759/cureus.42527

**Published:** 2023-07-27

**Authors:** Manish Goyal, Mayank Agarwal, Arun Goel

**Affiliations:** 1 Physiology, All India Institute of Medical Sciences, Bhubaneswar, IND; 2 Physiology, All India Institute of Medical Sciences, Raebareli, IND; 3 Physiology, All India Institute of Medical Sciences, Rishikesh, IND

**Keywords:** teaching materials, students, medical education, feedback, educational technology

## Abstract

Background and Objective

Multiple Choice Questions (MCQs) are commonly used in medical education for formative and summative assessment. However, the use of MCQs as a pedagogical tool in lectures is an area that is yet to be fully explored. This study aimed to gather feedback on including MCQs in lectures using an Online Audience Response System (OARS).

Methods

This quasi-experimental study involved 114 first professional MBBS students. A lecture with strategically integrated MCQs was delivered in a traditional classroom setting. Students answered the ten MCQs and provided feedback via OARS using their mobile phones. The feedback included eleven questions about student engagement, attentiveness, and critical thinking skills. Students' perception of the advantages and limitations of OARS in lectures was also collected. Data are presented as percentages and median with an interquartile range.

Results

Higher levels of engagement were reported by 80% of students; 81% felt improvement in understanding of the learning objectives and lecture content; 5% felt that mobile phone usage caused distractions; 79% reported increased focus and concentration; 84% reported that MCQs promoted their critical thinking skills; 75% reported enhanced overall learning experience without causing any discomfort; 69% believed that asking questions individually by the instructor is less effective than MCQs with OARS; 83% felt motivated to come prepared with study material when MCQs were included in the lecture; 67% preferred an even distribution of MCQs throughout the lecture; 53% preferred six to ten MCQs per lecture.

Conclusion

This study suggests that integrating MCQs in lectures using OARS can be a valuable pedagogical strategy in medical education and can potentially improve the learning experience by enhancing student engagement, attentiveness, and critical thinking skills. However, educators should also be aware of the potential limitations and take steps to mitigate them.

## Introduction

Medical education is a vital aspect of healthcare that aims to equip students with the necessary knowledge and skills to deliver high-quality patient care [1]. However, owing to the rising demand for qualified doctors to improve the doctor-population ratio, the increasing class size in medical colleges in India and Western countries has presented unique challenges that impact the effectiveness of medical education [2].

Lectures are a popular instructional method used to disseminate content to large groups of students. However, they can be passive and reduce critical thinking and problem-solving skills because of limited interaction between instructors and students [3-5]. Large class sizes negatively impact motivation, attention, and course content retention, making it logistically challenging to provide timely feedback and opportunities for active participation, discussion, and collaboration among students [6,7].

Incorporating interactive elements in lectures can be a practical and effective way to improve teaching and learning experiences, especially in large group settings [8-11]. Including questions in lectures can break the monotony of a traditional lecture format and add an element of surprise and challenge that engages the student to remain attentive and reduces the chances of boredom [12-14]. However, engaging all students in a large class setting is often impossible.

Multiple Choice Questions (MCQs) have emerged as a popular tool for assessing students in medical education. Incorporating MCQs into lectures has enhanced student engagement [15-17]. MCQs can improve interactions during lectures and make them more effective [18,19]. MCQs can be used for group discussions or peer-to-peer interactions, where students can discuss and debate options, exchange ideas, and learn from one another. According to one study, undergraduate students given five MCQs three to four days after attending a lecture significantly improved their ability to answer questions [20]. Another study found that discussion at the end of lectures was an excellent and effective revision tool [21]. Faculty development programs have also focused on improving MCQ item quality to enhance student's overall competency levels during yearly academic assessments [22].

MCQs can be quickly and easily administered, graded, and analyzed, making them a time-saving option for instructors who can focus on other teaching aspects and provide quality student feedback. Well-designed MCQs require students to apply their knowledge, analyze information, and decide based on the given options. Thus, MCQs can promote active student participation, increasing attention and a more dynamic and interactive learning experience [23]. Furthermore, MCQs can help students identify misconceptions or knowledge gaps, allowing for timely feedback and remediation.

In addition to MCQs, the Online Audience Response System (OARS) can further enhance the effectiveness of incorporating active learning strategies into large-group lectures [10]. These tools allow students to respond to MCQs in real-time during lectures, providing immediate feedback and potentially increasing engagement. OARs can also facilitate data collection for research purposes by measuring various learning outcomes such as engagement, motivation, participation, retention of course content, and student perceptions.

The present study investigated the effects of using OARS to collect MCQ responses incorporated in lectures on student learning experiences in large-group settings. A systematic study was conducted on first-year MBBS students in standard lecture theatre settings, collecting qualitative data to measure the impact of MCQs on various aspects of learning.

## Materials and methods

This quasi-experimental study involved the first professional MBBS students from a medical college after obtaining ethical clearance from the institutional review board (T/IM-NF/Physio/23/09 dated 15/0502023).

Ten MCQs were designed by the first author based on the learning objectives and study material. All three authors checked the face and content validity of the MCQs. These MCQs were strategically incorporated throughout a single lecture of an hour and distributed according to content relevance in a standard PowerPoint presentation format. Students were told a day before that the upcoming lecture would contain MCQs that they have to answer using OARS, but marks will not be a part of their formative assessment. MCQs were displayed on the screen at intervals during the lecture, and the students were encouraged to answer them using their smartphones through OARS. Students who did not use mobile devices were asked to write their answers in a notebook. After each MCQ, the results were promptly released, providing the students with immediate feedback on their comprehension of the material.

A structured questionnaire was developed to gather comprehensive feedback on the effectiveness of MCQs with OARS; the questionnaire aimed to elicit students' perceptions and experiences using this teaching method. All three authors checked the face and content validity of the questionnaire. The questionnaire consisted of 11 questions, nine close-ended with responses on a 5-point Likert scale (strongly agree, agree, neutral, disagree, and strongly disagree). One of the questions was close-ended, without any Likert scale options, while the remaining one was open-ended, allowing students to provide more detailed feedback.

Online responses from all participants were compiled in an Excel spreadsheet. Data collected from the feedback questionnaire were analyzed using descriptive statistics. Counts, percentages, and median with an interquartile range (IQR) were used to summarize the responses to the questions, providing a concise overview of students' perceptions and experiences.

## Results

A total of 114 responses were received from the class of 118 students. The questionnaire results used to gather feedback from the MBBS students on including MCQs in offline lectures using OARS revealed several key findings. 

Table 1 shows that incorporating MCQs with real-time responses using mobile devices has positively impacted students' engagement and understanding of the lecture content. Most students (80%) reported higher levels of engagement, while 81% felt that their understanding of the learning objectives and lecture content had improved. Furthermore, mobile devices for real-time response to MCQs improved students' attentiveness and concentration during lectures without causing significant distractions from smartphone usage. Most students (79%) felt this approach facilitated their focus and concentration during lectures. The students found MCQs challenging and stimulating, promoting their critical thinking skills (84%). Most students (75%) indicated that including MCQs using OARS enhanced their overall learning experience during lectures without causing any discomfort. Most students (69%) believed that asking questions individually by the instructor is less effective than MCQs with OARS for improving the learning experience.

**Table 1:** Feedback from students on the inclusion of MCQs with OARS in a lecture. IQR: Inter-Quartile Range; MCQs: Multiple Choice Questions; OARS: Online Audience Response System. The key for a 5-point Likert Scale: 1-strongly agree, 2-agree, 3-neutral, 4-disagree, and 5-strongly disagree. Due to rounding off, the sum of percentages may not add to the exact 100.

Number of responses = 114	Mean (IQR)	Strongly agree	Agree	Neutral	Disagree	Strongly disagree
MCQs helped you better understand the lecture content and learning objectives	1 (1-2)	70 (61%)	23 (20%)	17 (15%)	4 (4%)	0 (0%)
The inclusion of MCQs using the audience response online polling tool enhanced your engagement and participation in the lectures	1 (1-2)	58 (51%)	33 (29%)	17 (15%)	4 (4%)	2 (2%)
Using mobile devices for responding distracts you and decreases attentiveness and concentration during lectures.	4 (3-5)	1 (1%)	4 (4%)	32 (28%)	25 (22%)	52 (46%)
The MCQs were challenging and stimulating and facilitated your critical thinking skills	2 (1-2)	51 (45%)	44 (39%)	18 (16%)	1 (1%)	0 (0%)
The real-time response to MCQs using mobile devices improves your attentiveness and concentration during lectures.	2 (1-3)	40 (35%)	39 (34%)	24 (21%)	5 (4%)	6 (5%)
You were uncomfortable using the online audience response system to respond to MCQs during the lectures	4 (3-5)	1 (1%)	6 (5%)	23 (20%)	39 (34%)	45 (40%)
The inclusion of MCQs using the online audience response system enhanced your overall learning experience in the lectures	2 (1-3)	44 (39%)	41 (36%)	17 (15%)	8 (7%)	4 (4%)
Asking questions individually by the instructor is a more effective way as compared to MCQs with online audience response systems for improving the learning experience	4 (3-5)	1 (1%)	3 (3%)	31 (27%)	30 (26%)	49 (43%)
Including MCQs during lectures motivates you to come prepared with lecture material	1 (1-2)	59 (52%)	35 (31%)	18 (16%)	2 (2%)	0 (0%)

Additionally, most students (83%) felt motivated to come prepared with study material when MCQs were included in the lecture. Regarding MCQ spacing throughout the lecture, most students (67%) preferred an even distribution (Table 2). Feedback from open-ended responses revealed that about half of the students (53%) preferred six to ten MCQs per lecture (Table 3).

**Table 2:** Feedback from students on the placement of MCQs in a lecture MCQs, Multiple Choice Questions Due to round off the sum of percentages may not add to exact 100.

Number of responses = 114	At the end of the lecture	Before beginning the lecture	Evenly spaced throughout the lecture	Should not be asked
In your opinion, when do you think MCQs should be asked during the lecture.	19 (17%)	12 (11%)	76 (67%)	7 (6%)

**Table 3:** Feedback from students on the appropriate number of MCQs in a lecture MCQs, Multiple Choice Questions

Number of responses = 113	Up to 5	6-10	10-20
In your opinion how many MCQ are appropriate in one lecture (open-ended response)	16 (14%)	60 (53%)	37 (33%)

## Discussion

A questionnaire-based feedback survey conducted with MBBS first-year students indicated that integrating MCQs with OARS in lectures is a practical approach for enhancing students' engagement, understanding, and critical thinking skills, without causing significant distractions or discomfort. These findings provide insights into refining MCQs and OARS in medical education to promote active and reflective learning experiences.

The findings of this study align with those of previous research highlighting the benefits of using MCQs in medical education [15-17]. Positive feedback from students regarding increased engagement, improved attentiveness, and facilitation of critical thinking skills support the notion that MCQs can promote active learning and enhance students' participation in lectures. These findings are consistent with studies showing the effectiveness of incorporating interactive elements, such as MCQs, into lectures to promote deeper learning and knowledge retention [20].

The students well-received the use of OARS for real-time MCQs during the lecture. The convenience of using mobile devices for participation and the ability to receive immediate feedback contributed to high student engagement and satisfaction levels. This finding aligns with studies highlighting the advantages of using technology-enhanced tools for interactive learning in medical education [18,19].

Very few students reported being distracted (looking for web content or checking social media sites) by smartphone usage for answering MCQs during the lecture. A previous study explored the impact of smartphone use on classroom teaching and reported a positive perception by students [24]. With the rapid advancement in information technology, it is prudent to incorporate the advantages of technology in improving teaching and learning experiences [25].

The feedback from the students indicated that the MCQs were highly beneficial in reinforcing their understanding of the lecture content. Students appreciated how MCQs helped them review and consolidate their knowledge and encouraged them to participate actively in the learning process. In addition, some students provided valuable feedback and suggestions for improving the implementation of MCQs and OARS in lectures. Common suggestions include incorporating more challenging and diverse MCQs covering a more comprehensive range of topics to enhance critical thinking skills. Students also suggested aligning MCQs with the lecture content and learning objectives to facilitate better understanding and incorporating a greater variety of question formats, such as scenario- or application-based questions, to promote deeper learning. Providing immediate feedback on the correct answer after each MCQ rather than at the end of the lecture is also suggested to facilitate better learning. Finally, it was also suggested to incorporate graph- and image-based MCQs to increase lecture engagement.

These suggestions and feedback from students provide valuable insights for improving the implementation of MCQs and OARS in lectures, which can help refine the approach for future use. Additionally, open-ended responses from the students revealed that they appreciated using technology in the classroom to improve their learning experience. They found that MCQs helped identify knowledge gaps and promote self-assessment, thus contributing to a more active and reflective learning experience.

Limitations

Although this study has provided valuable insights into the potential benefits of MCQs and OARS, several limitations should be considered when interpreting the results. One potential limitation is the reliance on self-reported data, which may be subject to self-report, recall, or social desirability bias. Future studies should consider using objective measures of learning outcomes, such as pre-and post-lecture knowledge assessments or performance on standardized exams, to provide more robust evidence. Additionally, the sample size in this study was relatively small, and the study was conducted at a single center during a single lecture of an hour. Therefore, the generalizability of our findings is limited. Future research could replicate these findings with more extensive and diverse samples from multiple centers to improve the external validity of the results.

## Conclusions

The findings of this study suggest that MCQs and OARS can be valuable pedagogical tools for enhancing student engagement and learning during lectures. To optimize the use of MCQs and OARS in medical education, future research could explore how to design MCQs aligned with lecture content and incorporate diverse and challenging questions to promote higher-order thinking skills. Future research should also investigate the long-term impact of MCQs and OARS on knowledge retention and clinical performance. While further research is needed to strengthen the evidence base, the present study's findings suggest that these pedagogical tools can enhance student's learning experience in large group lectures and form instructional practices for promoting effective teaching and learning strategies in such settings.
